# Retropupillary Fixation of Iris-Claw Intraocular Lens for Aphakic Eyes in Children

**DOI:** 10.1371/journal.pone.0126614

**Published:** 2015-06-25

**Authors:** Martina Brandner, Sarah Thaler-Saliba, Sophie Plainer, Bertram Vidic, Yosuf El-Shabrawi, Navid Ardjomand

**Affiliations:** 1 Department of Ophthalmology, Medical University Graz, Graz, Austria; 2 Department of Ophthalmology, General Hospital Klagenfurt, Klagenfurt, Austria; Boston Children's Hospital, UNITED STATES

## Abstract

**Purpose:**

To report outcome, complications and safety of retropupillary fixated iris-claw intraocular lenses in a pediatric population.

**Design:**

Retrospective study.

**Patients and Methods:**

Ten consecutive pediatric patients (15 eyes) underwent placement of retropupillary fixated iris-claw intraocular lenses between October 2007 and July 2013 at the Department of Ophthalmology, Medical University Graz and General Hospital Klagenfurt, Austria. Postoperative visual acuity and complications were analyzed.

**Results:**

Median final best-corrected visual acuity improved by 0.12 logMAR from preoperative baseline. Mean postoperative spherical equivalent was -0.05 ± 1.76 D. No serious complications were observed intra- or postoperatively during the entire follow-up period of up to 40 months. One patient experienced a haptic disenclavation with IOL subluxation immediately after a car accident.

**Conclusion:**

Our study demonstrates that iris-claw intraocular lens implantation behind the iris is safe in children with lack of capsular support and yields excellent visual outcome with low complication rate.

## Introduction

The primary implantation of a hydrophobic intraocular lens (IOL) into the bag is the method of choice in children older than two years of age undergoing cataract surgery[1].

Such a procedure can only be performed, if the capsular bag is intact. If adequate capsular support is missing, the options of surgical refractive correction include anterior chamber IOLs, iris-sutured IOLs, scleral-sutured or intrascleral-glued IOLs[2–5].

Implantation of an IOL into the anterior chamber (ACIOL) can be associated with complications. If the IOL is undersized, corneal endothelial cell damage might happen due to IOL rotation, resulting in corneal decompensation. In cases of oversizing, the patient might develop iris ischemia, hyphema, iritis, secondary glaucoma or cystoid macular edema[2].

Suturing the IOL to the sclera or to the iris has the risk of suture breakage, resulting in IOL tilt or dislocation of the IOL into the vitreous. Patients with sclera sutured IOLs are also at a higher risk for vitreous hemorrhage and endophthalmitis[6].

The iris-claw IOL was developed in the seventies by Jan Worst for the correction of aphakia[7]. However, Siddiqui et al. have recently published data on iris-claw IOL fixation in the anterior chamber with a documented endothelial cell loss of 17% in children aged 8 to 15 years during a short follow up period of 12 months[8]. In a retrospective study with a follow-up period of up to 15 years Sminia et al. have shown that the endothelial cell count might decrease down to 1400 cells/mm^2^ in some eye, if the iris-claw IOL is located in the anterior chamber[9–11].

Rijneveld et al. described the option of retropupillary fixation of an iris-claw IOL in adults in 1994[12]. This technique has the advantage that the IOL is located behind the iris and therefore the negative influence on the corneal endothelium is less than with anterior chamber IOLs. This hypothesis was recently confirmed by Gonnermann et al. They have shown that the posterior enclavation of the iris-claw IOL has no significant influence on the corneal endothelial cell count[13, 14].

Herein we report on the posterior fixation of an iris-claw lens in children.

## Patients and Methods

The study was conducted in accordance with the tenets of the World Medical Association’s Declaration of Helsinki. Ethics Committee of the Medical University Graz has approved the data analysis of this retrospective clinical study. The authors had received written informed consent from the minor's parent before the operation. However, patients data were anonymized prior and de-identified prior to analysis.

### Patients data

This retrospective study comprised 15 eyes of 10 pediatric aphakic patients (8 male, 2 female; age ≤ 18 years) undergoing retropupillary iris-claw IOL (Artisan, Ophtec, Netherlands; Verisyse VRS54, Abbott, USA) implantation between October 2007 and July 2013. Eight patients (8/10, 80%) underwent surgery at the Department of Ophthalmology, Medical University of Graz and two patients (2/10, 20%) at the Department of Ophthalmology, General Hospital Klagenfurt. All patients were operated by one of three surgeons (BV, YE, NA) using the same technique. Three patients (6/15 eyes, 40%) with Marfan syndrome or homocystinuria and luxation of the crystalline lens underwent intracapsular cataract surgery with simultaneous retropupillary fixation of an iris-claw lens. All other eyes (9/15 eyes, 60%) were aphakic at the time of operation and underwent secondary IOL implantation. The IOL size was calculated in all eyes based on the horizontal white-to-white distance. A pediatric IOL with 7.5 mm diameter was implanted in one eye with a white-to-white distance of less than 10.5 mm. All children received a complete preoperative and postoperative ophthalmologic evaluation including age-appropriate assessment of visual acuity (Lea test, Snellen), cycloplegic retinoscopy, slitlamp examination, intraocular pressure measurements and dilated fundoscopy. Visual acuity was converted to logarithm of minimum angle of resolution (logMAR) values for statistical analysis.

Unilateral Amblyopia was defined as at least 2 Snellen lines difference in visual acuity between the eyes. Bilateral amblyopia was defined as bilateral subnormal best-corrected visual acuity (worse than 20/40).

IOL biometry and white-to-white measurement was performed using the IOL master (Zeiss Meditec, Germany) if the children were compliant and target refraction of + 0.5 spherical equivalent was planed in children 10 years of age or younger and emmetropia in children older than 10 years of age.

If the children were not compliant, the following refractive parameters were collected during an examination under anesthesia: Corneal topography (Keratron Scout, Optikon, Italy), axial length (contact A-scan ultrasonography) and horizontal white-to-white size (caliper, Katena, USA).

The corneal endothelial cell count could not be measured for all patients, since the cooperation was reduced in the young patients aged less than 9 years. We did not analyze this factor due to insufficient data for valid analysis.

IOL calculation was always performed with the SRK/T formula using an A-constant of 116.9 (manufacturers' recommendation for retropupillary fixation). This constant was always taken regardless of biometry mode (ultrasound or laser interferometry).

### Surgical technique

The operation technique was similar to that published by Wolter-Rössler et al. and Gonnerman et al.[13, 15]. Aphakic patients did not receive any dilating drops preoperatively. Three patients (6/15 eyes, 40%) underwent intracapsular cataract extraction before retropupillary IOL implantation. All operations were performed under general anaesthesia.

For retropupillary IOL implantation, two paracenthesis were performed at 2 and 10 o’clock or 3 and 9 o’clock. Miochol (Novartis, Germany) was afterwards injected into the anterior chamber, followed by Healon GV (Abbott, USA). The iris-claw lens was then inserted into the anterior chamber with the concave side toward the iris. The lens was held with a forceps at the optic (Implantation forceps 2-789-2, DK, UK), pushed behind the iris and fixed to the iris on the enclavation sites at the 3 and 9 o’clock position using a phakoemulsification spatula or the enclavation needle. An iridectomy was performed at the 12 o’clock position in the first 8 eyes (8/15, 53.3%), but not in the last 7 eyes (7/15, 46.7%). Viscoelastic was afterwards removed with the simcoe cannula, the corneoscleral incision closed with three to four nylon sutures (10–0). The conjunctiva was fixed with two 8–0 vicryl sutures at the end of the operation, before cefuroxime was injected into the anterior chamber.

Postoperative regime consisted of 1mg/kg methylprednisolone i.v. for 2 days, betamethasone drops hourly and gentamycin drops QID. Topical treatment was tapered off over the following 8 weeks.

Suture removal was performed three weeks to four months postoperatively depending on suture loosening.

## Results

The demographic data of the patients are listed in Table 1.

**Table 1:** Detailed summary of patient´s pre- and postoperative data.

Patient	Cause	Eye	Age	Preoperative	Lens	Amblyopia	Follow-up	Postoperative
(gender)			(years)^a^	DCVA	diameter^b^		(months)	DCVA
1(male)	Postsurgical aphakia	1	16	0.3	8.5	Y	23	0.3
2(female)	Homocystinuria	2	15	0.7	8.5	N	12	0
		3		0.4	8.5	N	12	0
3(male)	Trauma	4	9	1.0	8.5	Y	11	0.2
4(female)	Postsurgical aphakia	5	17	0.5	8.5	Y	40	0.5
		6		0.6	8.5	Y	40	0.6
5(male)	Marfan syndrome	7	3	0.4	7.5	N	19	0
		8		0.4	7.5	N	19	0
6(male)	Postsurgical aphakia	9	18	0.2	8.5	N	5	0.1
7(male)	Trauma	10	4	1.4	8.5	Y	12	0.2
8(female)	Trauma	11	8	0.8	8.5	Y	8	0.2
9(male)	Subluxatio lentis	12	5	0.5	8.5	Y	5	0.3
		13		0.4	8.5	Y	5	0.3
10(male)	Homocystinuria	14	17	1.0	8.5	y	6	1.0
		15		0.3	8.5	n	6	0

BCVA = best corrected visual acuity (logMar); Y = yes; N = no

^a^ Age at time of retropupillary iris-claw intraocular lens placement

^b^ Lens diameter in millimeters

Median preoperative best-corrected visual acuity (BCVA) was 0.64 ± 0.27 logMAR. The age of the patients at the time of IOL implantation was on average 11.2 ± 5.98 years.

Early postoperative mean refraction (spherical equivalent) was 0.4 ± 1.9 diopters (D). Final mean refraction was -0.05 ± 1.76 D with no astigmatism greater than 2.0D. Median final BCVA was 0.25 ± 0.21 logMAR. Two eyes of one patient showed postoperatively no increase of BCVA due to nystagmus and moderate amblyopia (Table 1, patient 4). Mean follow-up was 14.9 months (5 to 40 months).

An intraoperative complication occurred in two cases: one of the two enclavation sites of the IOL loosened during the aspiration of the viscoelastics and the IOL luxated partially into the vitreous. The lens was lifted with a 23G MVR blade angled (DORC, Netherlands) through a sclerotomy 3.5mm behind the limbus, grasped with the implantation forceps again and re-enclavated. The postoperative course was uneventful (patient 9, eye 12 and patient 10, eye 15).

**Fig 1:**
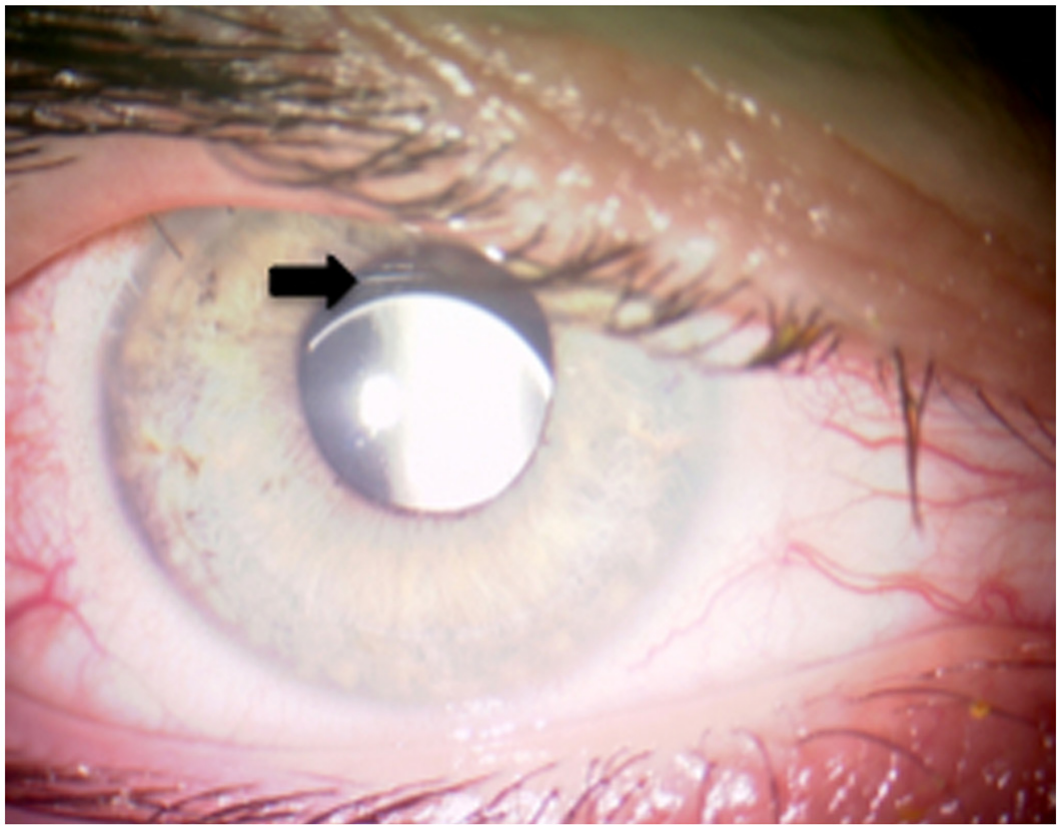
Postoperative photograph of IOL decentration. The picture shows mild downward decentration of the retropupillary IOL (patient 2, right eye, arrow).

Postoperatively, one eye (Fig 1) showed minimal IOL decentration requiring no repositioning since the patient had excellent visual acuity and did not complain about halos (patient 2). In two eyes of one patient intraocular pressure increased to 31 mmHg and 30 mmHg in the right and in the left eye, respectively, at the first postoperative day due to retained viscoelastic. Intraocular pressure decreased to 10 mmHg on both sides on the second postoperative day and stayed stable over the entire follow up period. One patient (patient 1) experienced a partial luxation of the IOL two years after implantation due to a car accident. The IOL was lifted from the vitreous through a sclerotomy with a 23G MVR blade and re-enclavated to the iris.

No patient developed macular edema or uveitis during the entire follow up period.

## Discussion

Surgical management of pediatric aphakia without adequate capsular support presents a surgical challenge. The implant has to last for several decades, stay centered and should not damage the adjacent tissue of the eye.

Surgically the easiest way for aphakic correction may be the implantation of an angle or iris supported ACIOL. Even if there have been some reports that angle supported ACIOL implantation is safe in children, there is always a concern for the corneal endothelium[2, 5]. Long-term follow-up of children with angle supported ACIOLs has additionally shown haptic migration and lens pigment deposits[5].

The main problem of the angle supported IOL is the sizing of the lens. In cases of undersizing, the IOL will rotate and damage the corneal endothelium and in cases of oversizing the patient will develop iris ischemia[16]. Pediatric eyes will still change in size and ACIOL sizing in this group of patients is therefore difficult and not perfectly possible.

Fixation of the IOL to the sclera has been the preferred choice for the last 20 years, especially the sutured sclera-fixed lenses. The main disadvantage of this technique is the long term complication of suture erosion or breakage and the dislocation of the IOL[6]. Buckley suggests the use of 9–0 instead of 10–0 prolene suture for children, since they might last longer, but his long term study showed a high incidence of IOL dislocation due to suture break up to 8 years after the operation[6].

The transscleral sutureless fixation of an IOL is a new technique, which is gaining popularity within the last few years[17–19]. Kumar et al. have recently published their data on his technique in children and shown that intra- and postoperative complications are low during a follow-up period of up to 36 months. However, this technique can be challenging, even for experienced surgeons[3].

The iris-claw IOL was originally developed for enclavation in the anterior chamber[7]. The lens has the advantage that one size fits all eyes if implanted in the anterior chamber. Anterior-fixation of the lens has been shown to be safe in children resulting in good visual outcome and low endothelial cell loss[8, 10, 20]. However, studies with long follow-up periods are missing and endothelial cell loss always remains a concern in eyes with anterior chamber IOLs.

**Fig 2:**
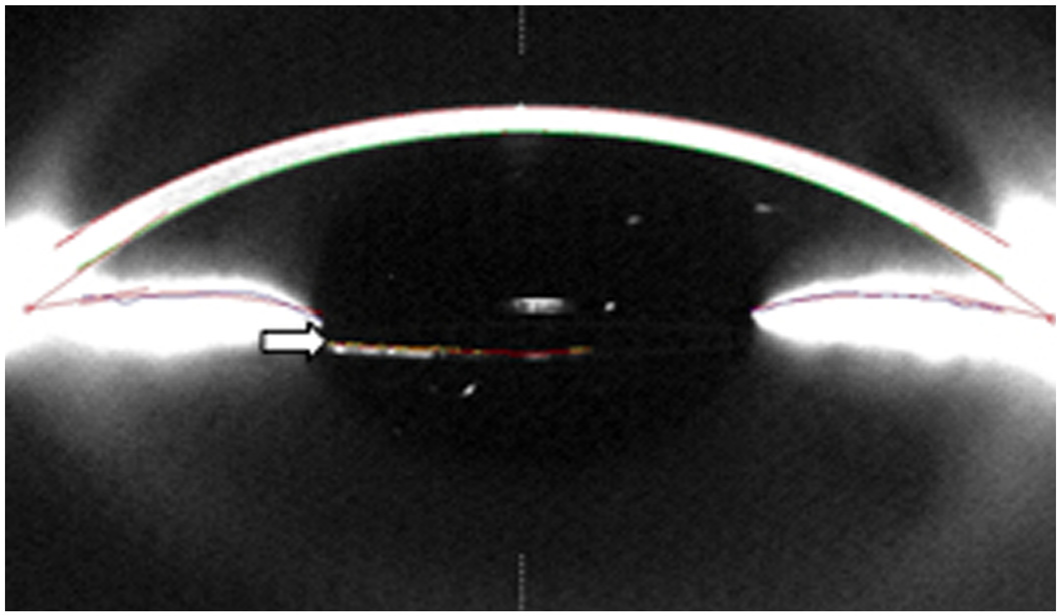
Pentacam image of a retropupillary fixated iris-claw lens. The picture shows the anterior segment with the IOL located just behind the iris (arrow). The IOL is located in a safe distance from the corneal endothelium

The posterior enclavation is technically more difficult, but is gaining high popularity within the last decade. It has the advantage of a better physiological intraocular refractive correction, a safer distance to the corneal endothelium (Fig 2) and is technically easier than suturing the lens to the sclera. Several studies have shown excellent results in terms of vision, postoperative complications and endothelial cell loss, after correcting aphakia with retropupillary implantation of an iris-claw IOL in adults[15, 21–23].

Gonnermann et al. have recently shown good results in children and adults with no significant effect on corneal endothelial cell loss during a mean follow-up period of 3 years[13, 14].

Measurement of endothelial cell count was difficult in our pediatric patients aged less than 9 years. We could get good acquisition quality of the endothelial cell count only in 8 eyes of 5 patients and due to lack of sufficient data, these results were not included in this study.

**Fig 3:**
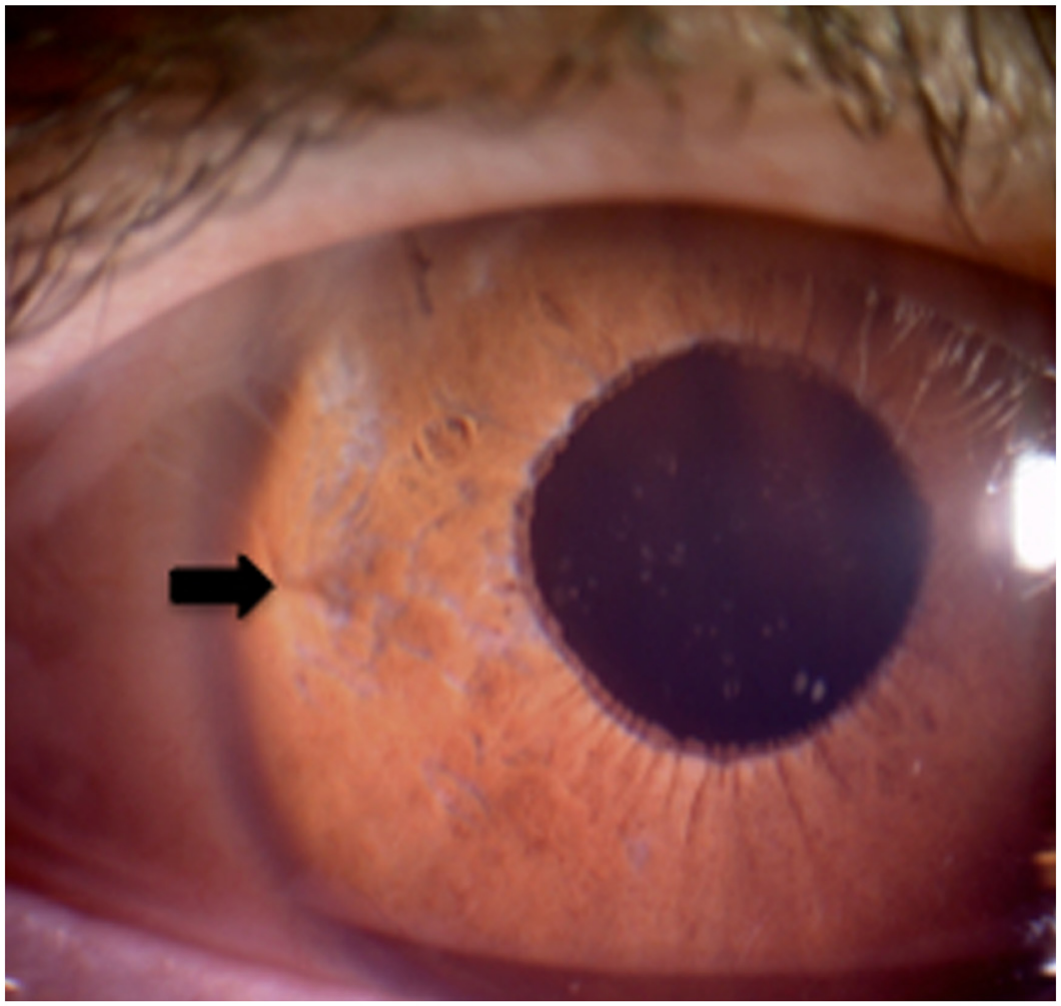
Postoperative photograph of a retropupillary fixated iris-claw lens after re-enclavation. The enclavation site of retropupillary fixated iris-claw lens is shown at the 9 o´clock position (arrow).

Macular edema or spontaneous IOL dislocation, as described in other studies, were not observed in our patients. However, one patient experienced an IOL disenclavation of one haptic immediately after a car accident, one year after initial surgery. The IOL was partially subluxated into the vitreous. The lens was lifted through a sclerotomy into the pupillary region and then reenclavated to the iris again (Fig 3). The patient did not experience any other postoperative complications. A similar case was reported by Gonnermann et al. and a coup-contrecoup mechanism of a blunt trauma is thought to be the pathophysiologic mechanism of the disenclavation[13].

Secondary glaucoma was observed in both eyes of one patient for one day due to retained viscoelastics, otherwise a significant increase of intraocular pressure was not seen in any patient during the entire follow up period.

Iridectomy was performed at the end of the operation between 2007 and 2010. Since there is some evidence that pupillary block cannot happen in eyes with a retropupillary fixation of an iris-claw IOL, we stopped that procedure afterwards. The Artisan IOL has a vaulted design and provides a clearance between the iris and the IOL. In cases of fixation behind the iris, the patient has additionally a deeper anterior chamber and iridectomy is not recommended in eyes with retropupillary fixation[13].

Retinal detachment, cystoid macular edema or endophthalmitis were not seen in any of our cases.

Our results are in accordance with previously published data[13], that the retropupillary fixation of an iris-claw IOL is a suitable technique to correct aphakia in children with low complication rate. However, longer follow-ups with a randomized study are necessary to confirm our preliminary results.
